# Progression of leprosy disability after discharge: is multidrug therapy enough?

**DOI:** 10.1111/tmi.12156

**Published:** 2013-08-13

**Authors:** Anna Maria Sales, Dayse Pereira Campos, Mariana Andrea Hacker, José Augusto da Costa Nery, Nádia Cristina Düppre, Emanuel Rangel, Euzenir Nunes Sarno, Maria Lucia Fernandes Penna

**Affiliations:** 1 Leprosy Laboratory, Oswaldo Cruz Institute, Oswaldo Cruz FoundationRio de Janeiro, Brazil; 2 Evandro Chagas Clinical Research Institute, Oswaldo Cruz FoundationRio de Janeiro, Brazil; 3 Department of Epidemiology and Biostatistics, Institute of Community Health, Fluminense Federal UniversityRio de Janeiro, Brazil

**Keywords:** disability grade, leprosy, risk factors, survival analysis, treatment

## Abstract

**Objective:**

To evaluate the risk factors related to worsening of physical disabilities after treatment discharge among patients with leprosy administered 12 consecutive monthly doses of multidrug therapy (MDT/WHO).

**Methods:**

Cohort study was carried out at the Leprosy Laboratory in Rio de Janeiro, Brazil. We evaluated patients with multibacillary leprosy treated (MDT/WHO) between 1997 and 2007. The Cox proportional hazards model was used to estimate the relationship between the onset of physical disabilities after release from treatment and epidemiological and clinical characteristics.

**Results:**

The total observation time period for the 368 patients was 1 570 person-years (PY), averaging 4.3 years per patient. The overall incidence rate of worsening of disability was 6.5/100 PY. Among those who began treatment with no disability, the incidence rate of physical disability was 4.5/100 PY. Among those who started treatment with Grade 1 or 2 disabilities, the incidence rate of deterioration was 10.5/100 PY. The survival analysis evidenced that when disability grade was 1, the risk was 1.61 (95% CI: 1.02–2.56), when disability was 2, the risk was 2.37 (95% CI 1.35–4.16), and when the number of skin lesions was 15 or more, an HR = 1.97 (95% CI: 1.07–3.63). Patients with neuritis showed a 65% increased risk of worsening of disability (HR = 1.65 [95% CI: 1.08–2.52]).

**Conclusion:**

Impairment at diagnosis was the main risk factor for neurological worsening after treatment/MDT. Early diagnosis and prompt treatment of reactional episodes remain the main means of preventing physical disabilities.

## Introduction

Leprosy is a major public health problem and epidemiological burden. It is a chronic infectious disease with acute and sometimes severe clinical presentations. In addition to its characterisation as an infectious transmissible disease, it also causes neurological disorders and, consequently, physical disabilities, which can occur at any stage of the disease even after the patient has been released from chemotherapy treatment and is considered bacteriologically cured.

The disease primarily affects the skin and peripheral nerve fibres. The pathogenesis of nerve damage is related to the presence of bacilli in Schwann cells, intraneural macrophages and the inflammatory processes arising from the immune response (Job 1994; Croft *et al.* 2000a; Scollard *et al.* 2006) despite the absence of identified bacillus in nerve biopsies. The damage to peripheral nerves results in sensory and motor impairment with characteristic deformities and disabilities. Studies show that approximately 20% of patients with leprosy present physical disabilities and social and psychological restrictions (Deepak 2003).

An evaluation of the rate of new leprosy cases in Brazil showed a rising trend over the last two decades of the 20th century. However, and most probably as a result of improved access to primary health care on the part of the population after the inauguration of the Unified Health System, (Penna *et al.* 2008) a decidedly downward trend began in the year 2000. The spatial distribution of leprosy shows great heterogeneity, with clusters of high-risk municipalities mainly located in the northern and midwestern regions of Brazil, in which leprosy disease is concentrated in certain geographical areas and among particular segments of the population. In the State of Rio de Janeiro, the site of this study, only 0.6% of the population inhabits what are considered high-risk clusters (Penna *et al.* 2009).

Early detection of new cases and treatment with multidrug therapy (MDT) are central to the disease control strategy recommended by WHO since 1991. One of the foremost priorities in the fight against leprosy is the avoidance of nerve damage (WHO 2011). Prevention of physical disabilities begins with early diagnosis of the disease, the recognition and treatment of such complications as neuritis and leprosy reactions, the identification of patients at risk of developing secondary complications, and timely intervention (WHO 2010).

One of the ways to evaluate the burden of leprosy is by measuring the frequency of neurological damage that can be attributed to the disease. Physical disability evaluation after treatment is highly important because disabilities represent the most pressing problem as they prevent patients from leading normal, productive lives. Furthermore, the presence of sequelae perpetuates the stigma of the disease. Preventing the development and/or progression of physical disabilities in patients during treatment and after discharge remains a major challenge.

The aim of this study was to evaluate the risk factors related to worsening of physical disabilities after the completion of therapy among patients treated with 12 doses of multidrug therapy (MDT)/WHO.

## Methods

### Design, settings and data collection

This cohort study was conducted at the Leprosy Outpatient Clinic, Leprosy Laboratory, Oswaldo Cruz Foundation (FIOCRUZ), Rio de Janeiro, RJ. The study was comprised of all patients with multibacillary (MB) leprosy who were registered at the clinic between October 1997 and December 2007, who formally agreed to participate and all completed the full treatment consisting of 12 supervised doses of the standard WHO multidrug therapy. These patients had been clinically diagnosed according to the Ridley and Joplin classification as Borderline-Borderline (BB), Borderline-Lepromatous (BL) and Lepromatous-Lepromatous (LL), as confirmed by skin smear and histopathological tests. The end date of the study was established as 31 July 2010; and the FIOCRUZ Research Ethics Committee approved the study.

All patients were submitted to dermatoneurological examinations, bacilloscopies to determine bacillary index (BI) and physical therapy evaluation to determine disability grade (DG) at diagnosis. Patients with multibacillary leprosy were monitored annually after release from treatment and underwent the same initial examinations.

Impairment status was determined by the maximum disability grade (DG) according to WHO (1998) criteria. The DG categories were as follows – 0: no eye, hand or foot problems due to leprosy; 1: decrease or loss of sensitivity in the eyes, hands and/or feet; and 2: visible deformity or damage in the eyes, hands and/or feet.

When the patient already presents nerve function impairment, the worsening of the condition cannot always be detected by the maximum grade of disability. For patients who completed treatment with some degree of impairment (DG = 1 or 2) in this study, the Bechelli disability index (Bechelli & Dominguez 1971) was also used. The Bechelli index counts each disability individually, ranging from a minimum 0.17 (=1/6) loss of sensitivity (Grade 1) in only one of the six evaluated sites, to a 9 (=54/6), which indicates that the patient is experiencing all the disabilities assessed in all six sites. This index provides a better option for evaluating the severity of and changes in disabilities. The outcome studied was the first worsening of disabilities after the patient completed therapy as measured by WHO DG and at any increase in the Bechelli index.

The following covariates were considered: socio-demographic variables such as: age, gender and educational level. Clinical variables studied were clinical presentation of leprosy as BB, BL or LL; the number of skin lesions at the beginning of treatment; oedema and cyanosis of the extremities at diagnosis; the initial bacillary index (BI); and initial physical disability (categorised as 0: no change; 1: abnormal sensitivity; and 2: visible deformities). Variables related to leprosy reactions included reactional episodes and the classification of types of reaction, that is, reversal reaction (RR), erythema nodosum leprosum (ENL) or neuritis. RR and ENL were confirmed by histopathological examination. Neuritis was defined as pain–whether spontaneous or at palpation in a peripheral nerve trunk–with or without nerve enlargement or function compromise. Neuritis was diagnosed when the neural symptoms occurred in the presence or not of reactional skin lesions.

The observation period begun at discharge from multidrug therapy and ended on the date of the first detection of a worsening in physical disability (event) or the date of the last visit for those under surveillance without worsening. Patients who reached the end of the study with no worsening of disability and those who were lost to follow-up (either due to death, transfer of health service or failing to return for annual consultations) were censored. The date of the occurrence of the event was considered the midpoint between the date of the previous assessment and the date of diagnosis of the event.

### Statistical analysis

The survival curve was constructed using the Kaplan–Meier (KM) method. The Cox proportional risk model was adopted to estimate the hazard ratio (HR) at 95% confidence intervals (CI 95%). The variables were organised hierarchically into blocks of variables; namely socio-demographics characteristics, clinical characteristics related to leprosy and leprosy reactions. In the construction of the model, the variables from each block that did not have a statistically significant effect on survival (*P* < 0.05) were removed one by one. After removing those variables that did not reach statistical significance in Model 1, the clinical variables were added (Model 2). This process continued until the inclusion of the last group of variables related to the reactional episodes was complete (Model 3), systematically adding the variables selected from the previous model to the new model. Statistical tests were considered significant at 5%. The proportionality of the risk of each variable was assessed using the Schoenfeld graph of standardised residuals. Statistical analyses were performed via statistical software R, version 2.6.2.

## Results

We evaluated 368 multibacillary patients who completed the WHO-recommended treatment of 12 supervised doses of MDT. During the study period, 103 events were detected after completing therapy. Seven patients died, five were transferred to other health services, and 73 did not return for their annual visit. The 180 patients who did not meet the final outcome criteria by the end of the study were considered censored on this date. The total observation time for the 368 patients was 1570 person-years, averaging 4.3 years of observation per patient.

Of the patients studied, 73% were male; 22.3% BB, 33.4% BL and 44.3% had the LL form. At the beginning of treatment, the mean age was 37.5 years (±16.2), ranging from 5 to 76 years. The average BI at the beginning of MDT was 2.7 + (±1.4), ranging from 0.16 to 5.33; and the BI at the end of treatment was 1.9 + (±1.5), ranging from 0 to 5.00. At the beginning of treatment, 221 (60.1%) patients had DG = 0; 104 (28.2%) patients had DG = 1; and 43 (11.7%) had DG = 2. Upon completion of treatment, 224 (60.9%) patients had DG = 0; 110 (29.9%) DG = 1; and 34 (9.2%), DG = 2. Table1 shows the frequency distribution of the variables under study.

**Table 1:** Frequency of the socio-demographic, clinical and reactional variables

Variables	Categories	*N* (%)	Events
Gender	Female	103 (28)	24
	Male	265 (72)	79
Age	0–15	14 (3.8)	1
	16–29	135 (36.7)	34
	30–39	70 (19)	22
	≥40	149 (40.5)	46
Education	≥11	47 (12.8)	10
	8–10	73 (19.8)	22
	<8	248 (67.4)	71
Clinical presentation	BB	82 (22.3)	22
	BL	123 (33.4)	40
	LL	163 (44.3)	41
Initial bacillary index	≤3	184 (50)	53
	>3	184 (50)	50
Initial disability grade	0	221 (60.1)	46
	1	104 (28.2)	37
	2	43 (11.7)	20
Cyanosis	Absent	163 (44.3)	35
	Present	205 (55.7)	68
Edema	Absent	176 (47.8)	47
	Present	192 (52.2)	56
Skin lesions	≤15	72 (19.6)	12
	>15	294 (79.9)	89
Leprosy reactions	No	72 (19.6)	11
	Yes	296 (80.4)	92
Reversal Reaction	No	259 (70.4)	63
	Yes	109 (29.6)	40
ENL	No	187 (50.8)	51
	Yes	181 (49.2)	52
Neuritis	No	209 (56.8)	39
	Yes	159 (43.2)	64

When the patient presented a physical disability at the completion of therapy, worsening of the disability was assessed by the disability index. There were 58 patients (15.8%) whose DG and index of disability worsened after the end of therapy, while 45 (12.2%) showed a worsened disability index alone.

Table2 shows impairment progression during MDT treatment. Among the 221 patients who began treatment with a DG = 0, 197 (89.2%) remained free of impairment at the end of treatment, whereas 24 (10.8%) developed some form of disability during treatment. Among those who began treatment with an impairment (DG = 1 or 2), 18.4% improved and 81.6% maintained some disability. Among the Grade 2 patients, 11.7% improved to Grade 0, 20.9% improved to Grade 1 and 67.4% maintained DG = 2.

**Table 2:** Disability Grade (OMS) evaluation between diagnosis and release from treatment

		End of treatment (*n*%)		
At diagnosis	*n*	0	1	2
0	221	197 (89)	24 (11)	0
1	104	22 (21)	77 (74)	5 (5)
2	43	5 (11)	9 (21)	34 (68)

For all the patients, the incidence rate of worsening of disability after discharge was 6.5/100 person-years. Among those who began treatment with no disability, the incidence rate was 4.5/100 person-years, while for those who started treatment with Grade 1 or 2 disabilities, it was 10.5/100 person-years.

**Fig 1:**
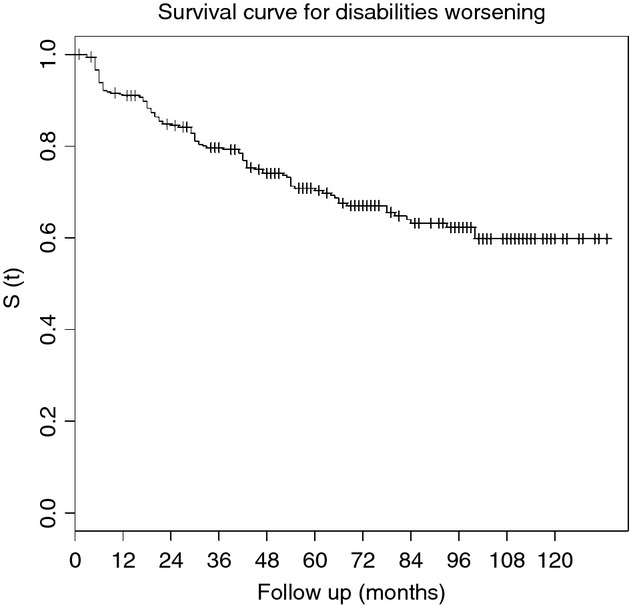
Survival analysis curve for worsening of disabilities among multibacillary patients treated with 12 doses of MDT/WHO.

At the end of the observation period, 40% of patients showed worsening of physical disability after discharge from therapy. The survival curve indicates that 9% of patients presented with worsened disability 1 year after completion of therapy and 30% presented worsening after 5 years (Figure1).

Table3 presents the results of the Cox model analysis. The bivariate analysis identified a significant association of the following variables: number of skin lesions, cyanosis, DG at diagnoses, leprosy reactions, RR and ENL. In the multivariate analysis, the variables of Model 1, that is, gender, age and educational level, were not associated with impairment after discharge. Nevertheless, due to their epidemiological relevance, gender and age were retained. We used the ‘change-in-point-estimate’ criteria regardless of the *P*-value of the statistical significance of the association.

**Table 3:** Risk of disability worsening after completion of therapy (HR) and 95% confidence intervals for the Cox model

			Model 1		Model 2		Model 3	
	Bivariate		Socio-demographic variables		Clinical variables		Reaction variables	
Variables	HR	95% IC	HR	95% IC	HR	95% IC	HR	95% IC
Socio-demographic variables								
Gender								
Female	1		1		1		1	
Male	1.29	0.81–2.03	1.28	0.81–2.02	1.08	0.67–1.72	1.11	0.69–1.76
Age (continuous)	1.01	0.99–1.02	1.01	0.99–1.02	1.00	0.99–1.01	1.00	0.99–1.02
Education (school years)								
≥11	1							
8–10	1.40	0.66–2.96						
<8	1.32	0.68–2.56						
Clinical variables								
Clinical forms								
BB	1							
BL	1.37	0.81–2.30						
LL	1.06	0.63–1.79						
Skin lesions								
Up to 15	1				1		1	
More than 15	2.01	1.10–3.68			2.06	1.12–3.78	1.97	1.07–3.63
Edema								
No	1							
Yes	1.13	0.77–1.67						
Cyanosis								
No	1							
Yes	1.72	1.14–2.59						
Initial bacillary index (continuous)	1.02	0.89–1.17						
Initial diasability grade					1			
0	1				1.82	1.16–2.86	1.61	1.02–2.56
1	2.03	1.32–3.14			2.77	1.60–4.77	2.37	1.35–4.16
2	2.80	1.65–4.74						
Reaction variables								
Reaction episodes								
No	1							
Yes	1.96	1.05–3.66						
Reversal reaction								
No	1							
Yes	1.55	1.04–2.31						
ENL								
No	1							
Yes	0.99	0.67–1.46						
Neuritis								
No	1					1		
Yes	1.90	1.28–2.84				1	1.65	1.08–2.52
Model results								
LR (loglik)				−545.74		−535.54		−532.71
*P*-value				0.061		0.000		0.017

LR, Likelihood ratio function test.

Among the clinical variables included in Model 2, initial grade of physical disability and number of skin lesions were significantly associated with worsening of disability. Patients with DG = 1 and DG = 2 showed a 1.82 and 2.77 times higher risk, respectively, than those without disability at baseline. The patients who had 15 or more skin lesions at baseline had a 2.06 times higher risk than those with <15. The presence of oedema and cyanosis of the extremities lost their significance. In Model 3, the initial DG and the number of skin lesions remained associated with worsening of disability. Among the variables related to reaction, the association between presence of reaction and type of reversal reaction lost their statistical significance when the model was adjusted for other variables. Patients with neuritis showed a 65% increase in the risk of worsening of disability after discharge, which was considered statistically significant (Table3). According to the Schoenfeld residual analysis, the risk of all variables was constant over time.

## Discussion

The duration of multidrug therapy (MDT) treatment of leprosy is considered the sole criterion for the cure of the disease, assuming that 12 months of MDT will be sufficient to induce a bacteriological cure (ILEP 1998). However, the neurological damage that leads to disabilities and deformities also occurs after therapy is complete. The impairment status worsened in 40% of all patients in the 10 years after release from treatment. This worsening was associated with the disability grade and the number of skin lesions at diagnosis and with the presence of neuritis. Thus, the worsening of leprosy impairment after specific treatment is principally related to late diagnosis, the extent and severity of the disease and the presence of neuritis.

In our study, the initial patient bacterial load was not associated with worsening of their disability grade. Although this variable was treated as continuous in the analysis presented, efforts have been made to test this association because the bacterial load could also indicate the extent of disease. For this reason, the BI was tested as a categorised variable (data not shown) in two classes (higher and lower than 3+) and then in three classes (up to 1.5+, 1.5 to 3+ and >3+). The initial BI was not associated with the worsening of disability after completion of therapy in any category. In this respect, our data is consistent with that of the Sharma *et al*. (1996) study, in which type of leprosy and BI did not affect the incidence, progression or regression of neural dysfunction.

Regarding the number of skin lesions, patients who had more than 15 skin lesions at baseline had twice the risk of worsening their disability after completion of therapy. This finding is consistent with that of Van Brakel and Khawas (1994), who evaluated risk factors for disability after treatment initiation using Cox regression, showing that the only variable that was in fact a risk factor was the extent of disease, characterised by the existence of more than 10 skin lesions, three or more affected nerves, and the involvement of more than two areas of the body. A study by Smith *et al*. (2009) examining MB patients without neurological damage at diagnosis showed that patients with more than 10 lesions had a 54% increased risk of developing new neurological injuries during and after treatment. Even so, these findings were not statistically significant (Smith *et al.* 2009).

Impairment at diagnosis was an important risk factor for neurological worsening after treatment; and patients who had a DG = 2 at diagnosis had a 2.37 times greater risk than those without disabilities. Several studies have demonstrated that DG at admission was the main risk factor for the development and evolution of impairments (Selvaraj *et al.* 1998; Meima *et al.* 1999; Croft *et al.* 2000b; Richardus *et al.* 2004; Kumar *et al.* 2012). A study conducted by Reed *et al*. (1997) included only MB patients treated with the aforementioned WHO/24-dose scheme and found that only initial DG = 1 (OR = 1.87) or 2 (OR = 1.98) remained independent risk factors for worsening of disability.

Our study showed the lack of association between RR and ENL and a worsening of disability among patients in treatment at a referral centre, that is, patients treated by specialists with access to corticosteroid therapy, which has a dramatic effect on immunoinflammatory regression. Overall, these results tend to suggest that the association between RR and ENL and disability worsening is dependent on the absence of proper clinical management.

Neuritis was treated with an initial dose of 1 mg/kg of prednisone for a month followed by a progressive 10 mg per monthly reduction over the remaining 5 months. Neuritis, despite also being treated with steroids, has proven to be a risk factor, probably because the inflammatory process occurs directly in the nerve itself and diagnosis customarily takes place sometime later. RR and ENL skin lesions lead patients to seek treatment earlier than complaints of neuritis, most likely when the pain is not as intense. Moreover, neuritis is also more difficult for health professionals to diagnose than RR or ENL, mainly due to the differential diagnosis required to detect neuropathic pain.

In this study, as in others, (Selvaraj *et al.* 1998; Meima *et al.* 1999; Croft *et al.* 2000c; Richardus *et al.* 2004; Gonçalves *et al*. 2009; Smith *et al.* 2009) gender was not a risk factor for worsening of disability. For example, in the studies carried out by Smith *et al*. (2009) and Croft *et al*. (2000b), both of which used Cox models, gender was not associated with neurological damage.

Age at diagnosis was also not a risk factor for worsening disability among our patients. The results presented in the literature are conflicting, however. Some studies show that older patients have a higher risk of developing new disabilities, (Selvaraj *et al.* 1998; Smith *et al.* 2009) which, conversely, is not observed in other studies (Croft *et al.* 2000b; Schuring *et al.* 2008; Kumar *et al.* 2012).

Our finding that 40% of patients had some degree of disability at diagnosis is similar to that of other studies (Schreuder 1998); (Ponnighaus 1995). A study in Nepal that assessed only MB patients stated that 56% presented with some disability at diagnosis (Reed *et al.* 1997). In other cohorts, MB patients also showed a high prevalence of disability upon admission (de Rijk *et al.* 1994; Croft *et al.* 2000b; Van Brakel *et al.* 2005).

The risk of disability at the end of a year was 9%. Our findings are similar to those resulting from assessing the risk of developing physical disabilities in MB patients treated with 24 doses of MDT, which showed a cumulative incidence of 10% for new disabilities during the 2 years of treatment (Reed *et al.* 1997). Another analysis found a higher risk of 21% (Meima *et al.* 2001). Our finding was similar to those of other studies that evaluated the dynamics of disability during treatment and after discharge and concluded that 79% of MB patients without neurological damage at diagnosis improved during treatment, whereas 84% remained unchanged after 60 months of follow-up (Meima *et al.* 2001; Richardus *et al.* 2004). Van Brakel *et al*. (2012) in a recent, field-based retrospective cohort study presented similar findings regarding physical impairment after MDT. On the other hand, a study of a small group of patients treated with either 24 or 12 doses of MDT showed no worsening of disability during therapy or after its completion (Escarabel *et al.* 2007).

As mentioned, ours was a prospective study to evaluate the therapeutic regimen of 12 doses of MDT by assessing the worsening of physical disability after discharge by examining a noteworthy sample followed by a long surveillance period. Because it was conducted in a referral centre, in which the criteria for assessment, diagnosis and treatment rigorously follow systematic operational protocols, the internal validity is maintained.

Conversely, the clinic assists referral patients whose health conditions are clinically severe and difficult to diagnosis. Therefore, our patients may not strictly represent the global population of patients with leprosy. The non-generalisability of disability prevalence and severity of the clinical condition does not apply to the risk factors involved in the observed associations. The associations of the variables considered risk factors and the outcome described are epidemiologically valid. Other studies (Van Brakel & Khawas 1994; Reed *et al.* 1997; Selvaraj *et al.* 1998; Smith *et al.* 2009) with similar findings are in agreement that these associations are biologically mediated.

As to the loss to follow-up inherent in cohort studies, in the present study, it can be stated that regarding the majority of variables, the patients lost to follow-up were not different from those who remained under observation (Table4). The follow-up losses for the most part occurred among patients with less severe clinical symptoms. In this case, then, the bias introduced is an underestimation of the association.

**Table 4:** Characteristics of patients who completed treatment versus those who lost follow-up

Variables	Categories	Total	Completed followup *n* (%)	Lost to followup *n* (%)	*P*
Gender	Fem	103 (28)	76 (26.9)	27 (31.8)	0.377
	Masc	265 (72)	207 (73.1)	58 (68.2)	
Age (years)	0–15	14 (3.8)	6 (2.1)	8 (9.4)	0.014
	16–29	135 (36.7)	103 (36.4)	32 (37.6)	
	30–39	70 (19)	58 (20.5)	12 (14.1)	
	40	149 (40.5)	116 (41.0)	33 (38.8)	
Education (years of schooling)	11	47 (12.8)	38 (13.4)	9 (10.6)	0.461
	8–10	73 (19.8)	59 (20.8)	14 (16.5)	
	<8	248 (67.4)	186 (65.7)	62 (72.9)	
Clinical forms	BB	82 (22.3)	57 (20.1)	25 (29.4)	0.131
	BL	123 (33.4)	94 (33.2)	29 (34.1)	
	LL	163 (44.3)	132 (46.6)	31 (36.5)	
Skin lesions	15	72 (19.6)	45 (16.0)	27 (31.8)	0.001
	>15	294 (79.9)	236 (84.0)	58 (68.2)	
Initial bacillary index	3	184 (50)	137 (48.4)	47 (55.3)	0.266
	>3	184 (50)	146 (51.6)	38 (44.7)	
Initial disability grade	0	221 (60.1)	169 (59.7)	52 (61.2)	0.934
	1	104 (28.3)	80 (28.3)	24 (28.2)	
	2	43 (11.7)	34 (12.0)	9 (10.6)	
Cyanosis	Absent	163 (44.3)	122 (43.1)	41 (48.2)	0.404
	Present	205 (55.7)	161 (56.9)	44 (51.8)	
Edema	Absent	176 (47.8)	131 (46.3)	45 (52.9)	0.282
	Present	192 (52.2)	152 (53.7)	40 (47.1)	
Reversal reaction	No	259 (70.4)	194 (68.6)	65 (76.5)	0.161
	Yes	109 (29.6)	89 (31.4)	20 (23.5)	
ENL	No	187 (50.8)	137 (48.4)	50 (58.8)	0.092
	Yes	181 (49.2)	146 (51.6)	35 (41.2)	
Neuritis	No	209 (56.8)	155 (54.8)	54 (63.5)	0.151
	Yes	159 (43.2)	128 (45.2)	31 (36.5)	

Physical disabilities still remain frequent, however, both at diagnosis and after treatment. Other considerations are necessary to both prevent disabilities and intervene in the evolution of existing disabilities. Early diagnosis and treatment of the disease and especially reactions remain the main means of preventing the development of physical disabilities. The value of better health education, capable of informing the general population and patients about the signs and symptoms of neural damage should not be underestimated, especially in terms of increasing the chances of early diagnosis. Disabilities are often overshadowed by social rejection and mental affliction caused by the stigma that persists in regard to leprosy in many if not most countries. Prevention of disabilities after treatment must receive more attention. Easy accessibility of health care can ensure a closely monitored follow-up of patients at most risk of developing disabilities after treatment and should also prevent worsening of disabilities. Patients without disabilities at discharge should be warned about the risk of future neurological impairments and learn how to recognise the first signs and symptoms. Follow-up of disabled patients with leprosy must be included in Leprosy Programmes; and these patients should immediately be referred to centres specialised in peripheral neuropathies.

The development of therapeutic studies focusing on reducing the development of disabilities after diagnosis and completion of therapy is of upmost important. And the technical quality of such studies must be ensured through appropriate sampling, sufficient monitoring and random participant allocation to different therapeutic groups.
